# Treatment of Croup

**Published:** 1856-05

**Authors:** 


### *Treatment of Croup.*

The following is extracted from an essay on the History, Nature and Treatment of Croup, by Albert Newman, M. D.—the Dissertation to which the Fisk Fund Prize was awarded, June, 1855.

The indications in the treatment of croup, are as follows:

I. To subdue the inflammation.
II. To moderate the laryngismus.
III. To prevent the formation of a false membrane.
IV. To produce the discharge of the false membrane when formed.
V. To support the patient's strength.

I. *Remedies for Subduing the Inflammation.*—In our selection of remedies for this purpose we shall be governed somewhat by the age of the patient and the general type of the disease. Whilst the treatment should in all cases be prompt and judiciously vigorous, the delicate age and susceptibilities of the patient ought never to be lost sight of. In sthenic cases of croup, where febrile action is high, and before it has been moderated by the occurrence of exudation, *bloodletting*, either general or local, may be resorted to. This remedy, however, is not appropriate in asthenic cases, nor in sthenic cases generally after the exudation has been poured out. Should the febrile action, however, continue high after exudation has occurred, indicating an extension of the inflammation downwards in the air-tubes, and the sthenic type of the disease be well-marked, the abstraction of blood may still be beneficial. The general fact, however, should be remembered that very young children bear the loss of blood badly. We would not advise *general* bloodletting in a patient under six years of age. If the patient be six years old or over, and robust and plethoric, and febrile excitement high, venesection may be resorted to with safety and advantage. In younger patients, where bloodletting is indicated by the severity of the symptoms and the sthenic character of the disease, it should be done locally by the application of leeches or cups. One or more cups may be applied between the shoulders, or to the nape; or two or three leeches to the top of the sternum or throat. In either case the quantity of blood taken should be carefully adapted to the age and condition of the patient. An ounce or an ounce and a half for each year the patient has completed will generally be borne. The value of this remedy has been variously estimated by different writers. With due regard to the character of the cases to which it is applied, we consider it a valuable remedy in fulfilling this indication; but neither general nor local bloodletting should be resorted to in any case in which they are not clearly indicated.

*Tartrate of Antimony.*—This is a remedy of great power and efficiency in fulfilling this indication. In many cases in which bloodletting would otherwise be indicated, this may be so administered as to form a safe and valuable substitute. It should be given in small doses, frequently repeated, so as to maintain a constant and steady sedative effect upon the circulation. It is generally proper to begin with doses of one-thirtieth to one-twentieth of a grain dissolved in water every hour or two, for a child one year old. For a child three or four years old the dose may be increased to one-fifteenth or one-tenth of a grain; the quantity given may be increased or diminished according to the effect produced. When administered in this way, its action upon the circulation is generally prompt and decided. The skin becomes cooler and the pulse slower and softer. The amount and frequency of the dose may be increased or diminished, as is found necessary to secure and maintain this effect.

In administering this remedy, it should be borne in mind that severe prostration is sometimes produced by it in very young children, and in those of a debilitated constitution. In all cases the patient should be carefully watched, and, upon the first appearance of prostration or diarrhœa, the medicine should be diminished or altogether withheld till the symptoms again indicate its repetition. It is not uncommon for diarrhœa to occur suddenly during its administration, and this diarrhœa it is sometimes exceedingly difficult to check. The stools are peculiarly watery and without odor. "An infant six months old has been destroyed by one-thirtieth of a grain of this remedy, and another barely escaped with life, after taking, in three doses, sixty drops of the hive syrup." (*Peaslee.*) In the majority of cases of sthenic croup, this remedy cautiously administered, as already advised, constitutes our main dependence, so far as internal medication is concerned, for arresting the inflammation. But it is not well borne in asthenic cases, and in those of a more sthenic type, after exudation has occurred and the febrile excitement somewhat subsided; it should generally be given in diminished doses. If vomiting is produced by the remedy, its sedative effect is diminished. We consider it, therefore, more efficient in controlling the inflammation when its administration is so regulated as not to produce this effect. The addition of opium in very small doses increases the tolerance of the medicine.

When a sufficient amount of the medicine to produce the desired effect upon the circulation is not borne by the stomach, and opium is not contraindicated, we are in the habit of adding one-half a drop, or more, in proportion to the age of the patient, of tincture of opium to each dose. The same addition may also be made when called for to diminish the action of the antimony on the bowels. This remedy has been used and recommended by some in very large doses in this disease. The largest quantity we have ever seen asserted to have been given, is in a case reported by Prof. Mettauer, of Baltimore, in the *Boston Medical and Surgical Journal*, Vol. XIV. To a child about nineteen months old, he gave, in a little more than two hours, the enormous amount of *half an ounce*, with as much ipecacuanha and antimonial wine.* Recovery followed; *post hoc, ergo propter hoc.* Prof. Mettauer remarks, and we think very appropriately, that this case "*furnishes new evidence of the astonishing resources of the infantile constitution, and its tenaciousness of life.*" We are no advocate for what is generally termed "*heroic treatment,*" and especially when the subjects are young children. Large doses of antimony are, in our opinion, unsafe and unnecessary in the treatment of this disease. The object is to produce and maintain a decided sedative impression upon the circulation without producing prostration. And this we believe to be best secured by administering the remedy in small and frequently repeated doses.

*Lobelia Inflata.*—When the tartrate of antimony, administered as above advised, causes troublesome diarrhœa, we have found the lobelia a valuable substitute. We believe it to approach more nearly in its operation to the tartrate of antimony than any other remedy we possess. It is a powerful remedy, and should be cautiously administered. We are in the habit of prescribing both the ethereal and the alcoholic tincture. Either may be given to a child one year old in doses of five or six drops every two hours, and the quantity or frequency of the dose may be increased or diminished according to the effect produced. With older children larger doses may be commenced with; but, in all cases, it is best to begin with moderate doses, and increase the quantity gradually, if necessary, to produce the desired sedative impression. Should symptoms of prostration be produced by the remedy, it must be at once withheld; and when the circulation again rises, smaller doses may be administered. It should not be given in asthenic cases.

*Nitrate of Potash.*—The sedative properties of the nitrate of potash are slight compared with those of the remedies already mentioned. It cannot, therefore, in any case be relied upon for arresting the inflammatory process. In asthenic cases, however, in which either of the preceding remedies would be deemed hazardous, and also in cases in which, from great irritability of the intestinal canal they cannot be borne, this remedy may be given; but rather as an auxiliary to remedics hereafter

*The following extract from Prof. Mettauer's "Case of Protracted Croup," shows the manner in which the remedy was administered. After describing the condition of the patient and the preliminary step of moistening the mouth, he says: "I next carefully introduced into the stomach, with a tea-spoon, a mixture composed of five grains of tartar emetic and ten of ipecacuanha, rendered semifluid with antimonial wine. This effort was finally successful, but the accomplishment of it was exceedingly difficult. A second dose of the medicines, mixed in the same manner, only augmenting the proportion of tartar to twenty grains, was quickly prepared and administered. After this attempt, a third and succeeding doses were prepared and administered in rapid succession; and I was now emboldened to give a heaping tea-spoonful of tartar at each dose, only employing the ipecacuanha and antimonial wine in quantities sufficient to suspend and render fluent so large a portion of the tartar. In this manner I proceeded, though not without much difficulty, and with fear and trembling lest the little patient might strangle, until the full contents of a half ounce vial of tartar emetic, and the same quantities of ipecacuanha and antimonial wine had been employed, requiring something more than two hours time for the accomplishment of this bold undertaking.—(*Boston Medical and Surgical Journal*, Vol. XIV. p. 102.)

to be noticed, than as a chief means of arresting the inflammation. It may be given in small doses of one or two grains dissolved in water, every two hours, to a child one year old.

*Cathartics.*—Early in the disease, if the bowels are not freely open, it is generally advisable to administer a gentle cathartic. By removing from the bowels all retained secretions and excretions, and, also, by relieving congestion of the portal circulation when present, we may expect to exert indirectly, a favorable influence upon the course of the disease. From three to five grains of calomel is, probably, the best cathartic that can be exhibited for this purpose. Should it not operate sufficiently, it may be followed in a few hours by castor oil.

*Emetics.*—An emetic administered at the commencement of the disease seems sometimes to have the effect of arresting it at once. It is only, however, in the forming stage of the disease that such an effect can be expected. After the disease is fully formed, the repeated exhibition of emetics can only do harm.

When an emetic is indicated, ipecacuanha may be used, combined, in sthenic cases, with tartrate of antimony, and, in asthenic cases, with sulphate of zinc. The syrupus scillæ compositus is an excellent article for this purpose in many cases. Thirty or forty minims may be given to a child two years old, and repeated every fifteen or twenty minutes till vomiting is produced.

*External Application of Cold.*—This is considered, by some writers, less beneficial than warm fomentations. Goelis considered it a dangerous application. We are aware that, to be beneficial, it must be continuously applied. Unless this point be secured, it will do more harm than good. Judiciously applied, however, we consider it a valuable aid in fulfilling this indication. We generally make the application by suspending a small pail, nearly filled with water, over the throat of the patient, and hanging a few small strips of cotton cloth over the edge of the pail so that one end may be immersed in the water. In this way, a continuous dropping is obtained. One, or, at most, two layers of cloth are laid upon the throat to receive it. Care should be taken that the patient's clothes and bed do not become wetted by the operation. If more convenient, the water may be constantly dropped from a sponge upon the throat. The compress upon which the water is dropped should be thin, that evaporation may go on rapidly.

*Application internally to the Seat of the Disease.*—Topical medication, in this disease, is of comparatively recent origin, not reaching back, at most, beyond fifteen years. Its great value and importance, however, are now fully established. The most efficient local application is, beyond doubt, a solution of the nitrate of silver. The value of this remedy as a local application in certain forms of inflammation has long been recognized and acknowledged by the medical profession generally. The first, we believe, to recommend its use in croup was M. Bretonneau. Guersent, Guiet, Bouchut, and other French practitioners, also recommended the same application. The strength of the solution used by M. Bretonneau was four grammes of the salt to thirty-two grammes of water; whilst M. Bouchut used ten grammes of the salt to thirty of water; and M. Guiet a still stronger solution. By all these writers the solution is directed to be applied to the throat and opening of the larynx only. The practicability and safety of introducing the solution *within the lar-*

*ynx* does not seem to have occurred to them. M. Bouchut, indeed, enjoins the greatest caution in making the application over the glottis, lest suffocation should be induced by too great a quantity of the liquid passing into the larynx.* The instrument used by the above writers for making the application, is a piece of bent whalebone, with a piece of fine sponge firmly attached to the end. M. Bretonneau directs the whalebone to be bent at five or six centimètres from its extremity at nearly a right angle.† Applied in this way at the commencement of the disease, there can be no doubt that many cases of croup may be at once arrested without passing the sponge into the cavity of the larynx at all. Generally, however, in order to secure the full benefit of the application, the sponge must be made to enter the larynx. Numerous and repeated experiments have now abundantly shown that the dangers, considered by M. Bouchut as attendant upon the application of this remedy to the fauces and opening of the larynx (*l'arrière bouche et la partie supérieure du larynx*,) do not exist; and that the application may be made, not only to these parts, but that the sponge may also be carried into the cavity of the larynx, and through and below the vocal cords, not only with perfect safety in every case, but with the most prompt and decided therapeutic effect. Dr. Horace Green was, we think, the first who conceived the idea of introducing the nitrate of silver within the larynx in this disease; and to him justly belongs the credit of having discovered and practically demonstrated both the safety of the operation and its great therapeutic value. He has also established the interesting fact, that "much less mechanical irritation is produced by the application of the nitrate of silver into the larynges of young children who are suffering from croup, than when it is introduced into those of adults who are affected with chronic disease of the larynx." The strength of the solution generally used is two scruples of the nitrate of silver in crystals to one ounce of distilled water. A solution of this strength is found to produce less irritation of the mucous membrane, and to exert a more beneficial effect than a weaker one. The application may be made by means of a laryngeal syringe, or a sponge probang like that recommended by M. Bretonneau. The latter is generally to be preferred, since it also answers another important indication hereafter to be mentioned, which the syringe does not answer. The application should first be made to the fauces and pharynx only; and then, after ten or fifteen minutes, the head of the child being held by an assistant, and the base of the tongue depressed with a spoon or some other instrument, the sponge, saturated with the solution, is carried over the

---

*Si la cauterization de l'arrie-bouche et de la partie superieure du larynx est advantageus, elle a aussi ses inconvenients qu'il faut connaitre pour tacher de les eviter. La suffocation immediate peut en etre la consequence, si l'on a laisse trop longtemps l'epunge sur la glotte, et si une trop grande quantite de liquide a penetre dans la larynx. Cet accident est fort grave, car il peut determiner la mort ou au moins la necessite de pratiquer aussito la tra tracheotomie.—(*Manuel Pratique des Maladies des Nouveaux-Nes, &c.—Par M. Bouchut.*)

†L'appareil est compose d'une eponge fine de la grosseur environ d'une noix, fixee au bout d'une baleine assez forte et recourbee, a la chaleur d'une bougie, a 5 ou 6 centimetres de son extremite et presque a angle droit. L'eponge est imbibee d'une solution de nitrate d'argent (au degree de 4 grammes de ce sel pour 32 grammes d'eau distillee.) Elle est introduite dans le fond de la gorge; l'epiglotte est soulevee et la solution exprimee ou-dessus de la glotte.—(*Formulaire Therapeutique, &c., concernant les Maladies de L'Enfrance.*)

top of the epiglottis, and pressed quickly downwards and forwards into the larynx. The sponge should not be more than one-third, or at most one-half inch in diameter for a child two years old or over; under this age, it should be somewhat smaller. The effect of the application of this remedy, when made early in the disease, is not infrequently to arrest the inflammation at once; and hence the importance of its early application, since, as already shown, the tendency of the inflammation is always to extend downwards in the air-tube. In every case of true croup, this application should be made *at our first visit;* and, if possible, over the whole extent of the inflamed membrane. The period at which the application should be repeated will vary in proportion to the severity of the disease and the effect produced. In the milder cases, once a day may be sufficient, while in those more severe it should be repeated three or more times daily. The operation is generally followed by cough, and a more or less free discharge of glairy and adhesive, or purulent matter, according to the stage of the disease; commingled frequently with shreds and patches of false membrane. Marked amelioration of the dyspnœa and cough almost uniformly follows. It is important that the operator be certain that the sponge really enters the larynx, for if he deceives himself upon this point the solution will be expressed into the œsophagus below the opening of the larynx, and of course no effect will be produced upon the disease. It is also desirable that the solution should, as near as possible, be made to reach the whole extent of the inflamed membrane.

"In most cases," says Dr. Horace Green, "it is only necessary to pass the sponge into the larynx; for the contraction of the glottis, which takes place on the introdaction of the instrument, presses the fluid from the sponge, which trickles down, and is diffused over the tracheal membrane." When, however, the inflammation extends to the bronchial tubes, it is desirable to pass the sponge through the rima glottidis, and into the trachea, if possible. In relation to the feasibility of this operation, Dr. Green* asserted, more than two years ago, that he had "succeeded in passing an armed probang down to the bifurcation of the trachea, probably over five hundred times;" and that employing "a probang nearly straight, armed with a sponge," he had pushed the instrument, not only down to the division of the trachea, but, turning it aside, *had "passed it at will, in many instances, into the right or left bronchus with as much ease and safety as the catheter is introduced into the bladder."*

II. *Remedies for Moderating the Laryngismus.*—The remedies already advised for arresting the inflammation will generally also, at the same time, fulfil this indication. The action of an *emetic,* especially in the early stage of the disease, is generally followed for a time by a marked diminution of the laryngismus. After the disease is fully formed, however, the frequent repetition of emetics for this purpose is not advisable or judicious. When we have reason to believe it due chiefly to irritating matters in the stomach, a suitable emetic may be administered, if not otherwise contraindicated. *Hydrocyanic acid* is a valuable remedy in fulfilling this indication, especially in the latter stages of the disease. Decided relief of the dyspnœa, cough, and restlessness, may often be obtained by its administration. The *application of nitrate of*

*See Braithwaite's Retrospect, Part XXVI. p. 60.

*silver* internally to the seat of the disease, as already advised under our first indication, is also an invaluable remedy for controlling the laryngismus in all stages and conditions of the disease. The *inhalation of watery vapor*, simple or medicated, with camphor, stramonium, or other narcotics, exerts also a favorable influence upon the laryngismus. Care should be taken, however, that they be not continued long enough, without intermission, to interfere with the already impeded aëration of the blood.

*Tracheotomy.*—The value and propriety of this operation in croup will be considered in another connection. It is conclusively shown, we think, by post-mortem appearances, that patients with croup do sometimes perish during a paroxysm of laryngismus from asphyxia *thereby induced.* In a case in which there seemed good reason to anticipate such a termination, notwithstanding the use of the remedies above advised, a resort to the operation would certainly be justifiable and proper.

III. *Means for preventing the Formation of a False Membrane.*—These may be included under two heads: 1. Remedies calculated to diminish the organizability of the exudation; 2. Means adapted to secure the removal of the exudation before the occurrence of organization. The remedies of most value under the first head are the preparations of mercury generally, of which calomel is much the most efficient. The value of this remedy in inflammations of serous membranes has long been an established fact in therapeutics. Its evident effect in these diseases is to promote absorption of the effusion, and to retard or prevent the formation of adhesions and false membranes. Absorption in these cases, is probably promoted rather by the direct effect of the mercurial upon the fibrin of the effusion than by any direct effect upon the absorbent vessels of the part. The direct effect of this class of remedies is probably to diminish the fibrin of the blood, and consequently that of the inflammatory exudation also. It may well be doubted whether the preparations of mercury have any effect beyond this in controlling either the progress or consequences of inflammation. But it is certain that they do produce this effect upon the plasma of the blood—*they diminish its organizability*. They are, therefore, valuable remedies not only in all inflammations of serous membranes, but also in all inflammations in which false membranes are liable to occur. For the purpose, then, of diminishing the organizability of the exuded plasma, small doses of calomel (¼ to 1 grain) may be given every fourth hour or oftener; diminishing the quantity if any marked cathartic effect is produced, or adding, if necessary, to each dose a small quantity of the pulvis ipecac. comp. It should be borne in mind, however, that this remedy is capable of doing much harm in cases to which it is not adapted, even in the small doses above mentioned. "It is now pretty generally known mercurial preparations (and especially calomel) are not well borne by scrofulous—and we will also add anæmic—subjects, whatever their ages or diseases. It produces great general debility; and if any accident exists requiring the aid of the reparative process, as an ulcer or a wound, it may indefinitely prevent this process from occurring, or even lead to phagedæna, or perhaps sloughing, in the part instead. The plasma always possessing a low grade of vitality (organizability) in such persons, becomes so far debilitated by the mercurial, that reparation is impossible; and it is only on discontinuing it, and substituting tonics and proper diet that the desired result ensues."* This condition of the blood-plasma in scrofulous and anæmic subjects, which prevents them from tolerating the action of calomel as just explained, diminishes also the probability of the organization of the exuded plasma in croup. If in any such case it is necessary to give any mercurial, the pil. hydrargyri, or some other preparation, should be preferred to calomel.

The *subsulphate of mercury*, or turpeth mineral, is a valuable substitute for calomel in such cases. It may be given in about the same doses that we would give calomel in sthenic cases. The time at which the administration of the mercurial is commenced is a matter of some importance. To produce its good effects, it must be administered before the exudation has become organized; and this is often early in the disease. In sthenic cases, therefore, we consider it advisable to commence its administration at once; waiting only to evacuate the bowels in cases in which a cathartic is indicated.

*Alkalies.*—This class of remedies also diminishes the organizability of the plasma of the blood. Their effect in this way, however, is much less than that produced by mercurials. They may be given in asthenic cases in which mercurials are inadmissible; and in such cases they constitute a valuable substitute for preventing the formation of a false membrane. The carbonate or bicarbonate of soda, or a corresponding salt of potassa, may be given in doses of from three to five grains to a child one year old, and repeated every fourth hour. We generally prefer the bicarbonate of soda. In the later stages of the disease, when a stimulant effect is also desired, the *bicarbonate of ammonia* may be administered with advantage. By diminishing the fibrin of the exudation they also favor expectoration, and thus contribute the second end to be secured under this indication, viz: to secure the removal of the exudation before organization has occurred.

The remedy which has perhaps been most relied upon for this purpose, is *emetics* frequently repeated. These have been given to an extent and with a recklessness almost incredible. A careful examination of the mechanism of vomiting, we think will show the complete impossibility of any matters contained in the air-tubes being ejected therefrom during the continuance of that act.† Carpenter says the act of vomiting "bears great resemblance to the act of coughing, differing chiefly in this, that in vomiting *the larynx is closed during the whole operation*, whilst it is only closed momentarily in coughing."‡ In both, the first act is a full inspiration, by which the thorax is distended, the diaphragm becoming at the same time strongly contracted, and the rima glottidis closed; thus preventing the escape of the air from the air-passages. Next, in coughing, the rima glottidis is suddenly opened, and the abdominal muscles contracted forcibly, whilst the diaphragm at the same time relaxes; the lungs are thus suddenly compressed, and the contained air forcibly expelled from the air-passages. In vomiting, also, a full inspiration is first

*Prof. Peaslee's Monograph on Croup, p. 23.

†For an excellent argument on this point, see Prof. Peaslee's valuable and original Monograph on Croup, to which we acknowledge our indebtedness for many ideas and suggestions which we have seen nowhere else.

‡Carpenter's Principles of Human Physiology, 3d ed., p. 385.

taken, the diaphragm contracted, and the rima glottidis closed, as before. Next, the abdominal muscles contract suddenly and forcibly, *but the rima glottidis remains closed, and the diaphragm strongly contracted.* The stomach is thus compressed between the contracted and depressed diaphragm and the abdominal muscles, and its contents expelled. But since the rima glottidis is closed, and the diaphragm contracted during the whole act, it is evident that nothing can be expelled from the air passages. The use of emetics, therefore, for this purpose, is neither rational or justifiable, since much injury is liable to result from them.

*Expectorants.*—The alkalies have already been mentioned as favoring expectoration by thinning the exuded plasma. After febrile action has subsided, the syrup of senega, or of squill, may be given for the purpose of increasing the expectoration, and thus aiding in detaching and removing the exudation.

*Counter-irritants.*—These are supposed to favor the absorption of the exuded plasma, and, in this way, they may have some effect in preventing its organization. After febrile action has somewhat subsided, stimulating liniments or blisters may be applied for this purpose.

*The Sponge Probang.*—The application of this instrument saturated with a solution of the nitrate of silver, as already advised for subduing the inflammation, is also by far the most efficient means for securing the removal of the exudation. More or less of this exudation is usually removed directly by the sponge, and the application is generally followed by cough and a considerable amount of expectoration. The solution, moreover, destroys the organizability of the exudation, and aids in the disorganization and detachment of the false membrane when formed.

IV. *Means for Producing the Discharge of the False Membrane when formed.*—These are precisely the same as those specified under the last preceding indication for securing the removal of the exudation before organization has occurred; the most important of which is the *sponge probang.* The sponge should be carried into the trachea; and the operation repeated as often as the urgency of the case requires. The presence of the false membrane may not be as already stated, the only or the chief source of danger in this disease; nor its removal from the air-passage, however effected, always followed by recovery. When present, however, it must greatly increase the danger by impeding respiration, both directly by its bulk blocking up its air-passages, and indirectly by increasing the laryngismus. Its speedy removal, however, is an object of the greatest importance. We would, therefore, urge the importance, in all cases in which there is reason to suspect the existence of a false membrane, of a thorough application of this remedy for this purpose. A considerable number of cases are now on record in which the life of the patient has evidently been saved in this way. The sponge should be passed through the rima glottidis, and as low into the trachea as possible. This remedy, if it were not more efficient for this purpose than emetics, has certainly the great advantage over them of being entirely harmless in all stages and types of the disease. Emetics at this stage of the disease are generally entirely out of place, and ought *never* to be given for the purpose of removing the false membrane.

*Tracheotomy.*—The opinions of medical writers of the value of this operation in croup are various and contradictory; and, under existing circumstances, it is perhaps impossible to form a just estimate of its value. When resorted to, it has generally been only in the last and hopeless stage of the disease, when little benefit could be expected from any remedy. There is much truth in the observation of Louis, that as "long as bronchotomy is considered an extreme measure *(un dernier ressort,)* it will be always performed too late." It is evident that a correct estimate of the value of the operation can only be formed from a consideration of its results when seasonably performed. Considering the unpromising circumstances under which the operation has been generally resorted to, its results have been, on the whole, sufficiently favorable, in our view, to justify its continuance at least. We would, however, insist upon the importance—for the reputation of the operation as well as safety of the patient—of not delaying the operation in cases in which it is actually required, till all reasonable prospect of benefit is passed. The operation is said to have been much more successful in France than in England. This is accounted for partly, if not wholly, by the fact that the operation is performed earlier in the former than in the latter country. A judicious and persevering use of the local application of the nitrate of silver will no doubt diminish much the number of cases in which the operation would be otherwise required. In cases which have terminated fatally after the use of the sponge probang, it has been found that the false membrane was completely removed as low down as the sponge was passed.

It should be borne in mind that the object of the operation is not solely to allow more air to pass into the lungs than is admitted through the constricted rima glottidis. In cases in which a false membrane forms, the disease generally spreads rapidly to trachea and also to the bronchi. In such cases, the benefit to be derived from tracheotomy is twofold:

1. It enables us to aid in removing the false membrane, through the opening made in the trachea by mechanical means.

2. It enables us to apply topical remedies, such as the solution of nitrate of silver, through the same opening directly to the trachea and bronchial tubes. Trousseau, who has probably operated more times and with better success than any other surgeon, follows the operation with the application of the nitrate of silver in this way. And Prof. Peaslee thinks that circumstances may exist which would justify the operation for this purpose alone. Upon this point we may be pardoned for again quoting from his valuable Monograph on Croup. He says (p. 29:) "But we may make an opening for the sake of making applications through it to the larynx, trachea, and bronchial tubes; and here an entirely new field opens to us. And we hope not to startle those overmuch who shudder at the idea, both of the operation and of the probang, now so frequently mentioned, if we say that, in our opinion, cases may occur in which it is justifiable to make an opening into the trachea for the mere purpose of applying the caustic solution and other remedies, directly to the seat of the disease, and to the extent required. If, for instance, the case be sthenic one, while from spasm or swelling of the larynx, or some other cause, it is found impossible to pass the caustic solution through the rima glottidis, though it is very certain that a false membrane will be, or has formed, and all of several children of the same family attacked by the disease have died of it; in this combination of circumstances, certainly we should feel justified in performing the operation for the object now under consideration." In regard to the time at which the operation should be resorted to, no rule can be given. It should be performed, if at all, as soon as we become satisfied that other means will not prevent a fatal termination, and before the vital powers are exhausted through want of a due supply of aërated blood; whether it be at the end of twelve hours or a week. After the disease has extended to the smaller subdivisions of the bronchial tubes, and they have become to a considerable extent blocked up by the exudation, little benefit can of course be expected from the operation.

V. *Means for Supporting the Patient's Strength.*—In the later stages of the disease, and throughout the course of the more asthenic cases, this is an important indication. It is to be borne in mind that the false membrane, when present, will be spontaneously dispatched and removed, provided the patient's strength holds out sufficiently long. We would, therefore, as far as possible, husband the patient's strength by the avoidance of all unnecessary medication, especially of powerful and prostrating emetics; and by the administration of suitable nourishment, adding, as the strength begins to fail, stimulants and tonics. Carbonate of ammonia, wine-whey, weak milk punch, or egg beat with wine may be used. Where a more permanent effect seems desirable, tonics may be also administered.

*Summary of Treatment.*—When called to see a case of croup during the first paroxysm of the laryngismus, it is generally proper to administer at once, if it has not been done before, a suitable emetic. We are in the habit of using for this purpose, more frequently than anything else, the syrupus scillæ compositus. To a child, two years old, this may be given in doses of half a tea-spoonful, and repeated every fifteen or twenty minutes till vomiting is produced. In cases decidedly asthenic, we prefer a combination of ipecac and sulphate of zinc; and in sthenic cases, the tartrate of antimony, alone, or combined with ipecac, may be given instead of the above preparation, if preferred. The action of the emetic at this stage of the disease is generally followed by marked relief of all the urgent symptoms. The improvement, however, is rather apparent than real, and depends entirely upon a temporary relief of the laryngismus. Our remedies must now be directed vigorously to arresting the inflammation. Especially should it be borne in mind that the earlier, judicious, and vigorous treatment is instituted, the more prompt and beneficial will be its effects. Whilst the inflammation is confined chiefly to the larynx, we have it in our power, in addition to the general treatment, to apply local remedies to the whole of the diseased surface. This favorable period, should, in no case, be allowed to pass unimproved. Experience has fully shown the danger and irrecoverable loss of delay. After the administration of the emetic, therefore, we would proceed at once to apply a solution of the nitrate of silver to the mucous membrane of the larynx with the sponge probang. This should not be omitted in any case of actual croup. If the patient be robust and plethoric, the dyspnœa considerable, and the pulse very frequent, it is generally advisable to apply cups or leeches in the manner already advised; or, if the patient be over six years old, to take blood from the arm. In milder cases, however, this remedy may be safely omitted. In cases in which it is required, a cathartic dose of calomel may next be administered; and afterwards, except in cases decidedly asthenic, the patient should take small doses of tartrate of antimony every hour, or every two hours, according to the urgency of the symptoms. The amount and frequency of the dose should be so regulated, if possible, as to produce a decided impression upon the character and frequency of the pulse, without causing vomiting. At this stage of the disease, this is altogether the most important internal remedy we possess. At the same time, also, we generally order small doses of calomel every four hours. In the severer cases, cold water may, at the same time, be applied to the throat in the manner already advised.

In asthenic cases, in which the tartrate of antimony is not admissible, the nitrate of potash may be freely given in solution instead; and if any mercurial is considered necessary, the turpeth mineral, or pil. hydrargyri, should be substituted for the calomel. In such cases, local applications of the nitrate of silver must constitute our main dependence. They should, therefore, be made with the utmost thoroughness, if possible, over the whole extent of the inflamed membrane, and repeated as often as the symptoms indicate. If repeated every two hours, it will do no harm. In many cases these remedies will arrest the disease at once. It is not, indeed, unusual to see cases of considerable severity arrested within twenty-four hours by the sponge probang and tartrate of antimony alone. Should the disease, however, continue, notwithstanding the use of the above means, the application of the nitrate of silver should be repeated, and the tartrate of antimony continued in doses proportionate to the frequency and hardness of the pulse and the heat of the skin. Should the antimony cause diarrhœa or otherwise disagree with the patient, the lobelia may be substituted. If the laryngismus is severe, the patient may also be allowed to breathe the vapor of hot water, simple or medicated, with camphor or stramonium, or some other narcotic; but for a few minutes only at a time. The mercurial, in cases to which it is adapted, may also be continued; or in asthenic cases, the bicarbonate of soda, or some other alkali, may be administered with a view to diminish the organizability of the exudation. Should there be evidence in the progress of the disease of the formation of a false membrane in the larynx and trachea, the sponge probang should be more frequently and perseveringly used. When the heat of the skin has subsided, some stimulating liniment may be applied to the throat, and the cold water, if previously applied, omitted. At this time, also, the senega or squill may be administered with benefit. Should the disease, however, progress, notwithstanding the above treatment, including the frequently repeated and thorough application of the nitrate of silver to the seat of the disease, and the lips become livid or purple, the dyspnœa great, and signs of exhaustion begin to appear, the tartrate of antimony and nitrate of potash should be at once omitted and stimulants cautiously substituted. At the same time, the operation of tracheotomy may be considered. The indications for this operation, and the objects to be secured by it, have already been sufficiently discussed, and need not be here repeated.—*Monthly Stethoscope & Reporter.*

